# Effects of Alpha-Ketoglutarate Supplementation on Growth Performance, Diarrhea Incidence, Plasma Amino Acid, and Nutrient Digestibility in Weaned Piglets

**DOI:** 10.3390/ani15121723

**Published:** 2025-06-11

**Authors:** Weiyan Sun, Ruyi Han, Hongbo Xi, Wenning Chen, Yanpin Li, Qingchao Zhou, Xilong Li, Kaikun Huang, Valentino Bontempo, Xu Gu, Xianren Jiang

**Affiliations:** 1 Key Laboratory of Feed Biotechnology of Ministry of Agriculture and Rural Affairs, Institute of Feed Research, Chinese Academy of Agricultural Sciences, Beijing 100081, China; swy001103@163.com (W.S.); hhhry5201314@163.com (R.H.); liyanpin@caas.cn (Y.L.); lixilong@caas.cn (X.L.); guxu@caas.cn (X.G.); 2 Shenzhen XinTianhe Biotechnology Co., Ltd., Shenzhen 518000, China; xihongbo@kexing.com (H.X.); huangkaikun@kexing.com (K.H.); 3 Department of Veterinary Medicine and Animal Science (DIVAS), University of Milan, 26900 Lodi, Italy; wenning.chen@unimi.it (W.C.), valentino.bontempo@unimi.it (V.B.); 4 Hebei Kangda Livestock and Poultry Breeding Co., Ltd., Langfang 065000, China; 15369860606@163.com

**Keywords:** piglets, feed additive, gut health, weaning, short-chain keto acid

## Abstract

Alpha-ketoglutaric acid (AKG) is a key intermediate in the tricarboxylic acid cycle and plays a crucial role in energy production and amino acid metabolism. This study aimed to evaluate the effects of AKG on growth performance, nutrient digestibility, plasma biochemical parameters, and plasma amino acid profiles in weaned piglets. A total of 72 weaned piglets with an average weight of 7.33 kg (±0.50 kg) and an average age of 28 (±2 days) were randomly assigned to 3 dietary treatments with 6 replicates per group in a 42-day trial. The treatments included a basal diet (CT), a basal diet with 500 g/t AKG (AKG1), and a basal diet with 1000 g/t AKG (AKG2). Blood samples were collected on days 14 and 42, and fecal samples were collected on day 42. The results showed that diets including 500 g/t and 1000 g/t AKG significantly reduced diarrhea incidence in piglets compared to the CT group (*p* < 0.01). Moreover, diets including 1000 g/t AKG enhanced fecal dry matter digestibility, plasma albumin (ALB), and glucose (GLU) concentrations on day 42 compared to the AKG1 group (*p* < 0.05). In conclusion, diets including AKG reduced diarrhea incidence in weaned piglets, potentially through improved nutrient digestibility and enhanced systemic health. The 1000 g/t level exhibited greater beneficial effects than 500 g/t.

## Simple Summary

The post-weaning period is critical for piglet growth and directly affects production efficiency in swine farming. Alpha-ketoglutaric acid (AKG) has shown potential in animal nutrition due to its role in energy metabolism, antioxidant defense, anti-inflammatory activity, and immune regulation. In this study, AKG supplementation significantly reduced the incidence of diarrhea, improved nutrient digestibility and amino acid utilization, and contributed to better overall health in weaned piglets.

## 1. Introduction

Alpha-ketoglutarate (AKG) is a short-chain, non-toxic carboxylic acid with high stability in aqueous solutions. In biological systems, AKG serves as a crucial intermediate in the tricarboxylic acid (TCA) cycle. It is generated through the oxidative decarboxylation of isocitrate by isocitrate dehydrogenase and the oxidative deamination of glutamate by glutamate dehydrogenase, thereby playing a pivotal role in bridging carbon and nitrogen metabolism [1]. Furthermore, AKG serves as a precursor for the synthesis of L-glutamate, L-glutamine, L-proline, and L-arginine in various tissues, bridging carbohydrate and nitrogen metabolism and contributing to amino acid homeostasis and ammonia detoxification [2,3,4]. AKG facilitates the oxidation of nutrients such as amino acids, glucose, and fatty acids to generate cellular energy [5]. In particular, extracellular AKG serves as an important energy source for gastrointestinal cells. It is also recognized as a key molecule in regulating nitrogen transport, protein metabolism, gene expression, and the cellular redox state [6,7].

The biological functions of AKG extend to the regulation of protein synthesis, muscle and bone development, immune system homeostasis, and glucose and lipid metabolism. For example, AKG can inhibit glutamine degradation and activate the mammalian target of the rapamycin (mTOR) pathway to promote protein synthesis in porcine intestinal epithelial cells [8]. It also induces the phosphorylation of JNK, mTOR, S6K1, and S6, thereby promoting osteoblast differentiation [9]. Andersen et al. showed that dietary AKG supplementation in piglets enhances bone length, ultimate strength, and maximum elastic strength [10]. Furthermore, it can inhibit glucose-6-phosphate synthesis, reduce plasma leucine concentrations, and enhance nitric oxide production in endothelial cells, thus improving insulin sensitivity [11]. AKG is also involved in carnitine synthesis, facilitating mitochondrial β-oxidation of fatty acids, and promoting lipid degradation [12,13]. Previous studies demonstrated that dietary AKG supplementation alleviated lipopolysaccharide (LPS)-induced liver histopathological damage and improved liver amino acid profiles in weanling piglets [14].

Glutamine exhibits limitations such as poor stability and low solubility and can generate toxic by-products such as pyroglutamic acid and ammonia, which limit its application in animal production [15]. Dietary AKG supplementation has been reported to enhance glutamine metabolism and amino acid utilization in intestinal cells [16]. Moreover, AKG has been shown to promote bone growth and improve both apparent total tract digestibility (ATTD) and apparent ileal digestibility (AID) of phosphorus and calcium in the intestines of piglets [17]. It also alleviates intestinal inflammation, enhances epithelial repair under stress, and supports gut health during early weaning [18]. Additionally, AKG supplementation modulates the intestinal microbiota by promoting probiotic growth, increasing butyrate and valeric acid concentrations, and reducing intestinal ammonia levels in growing pigs [19]. Studies have shown that AKG can attenuate inflammation by suppressing the activation of M1 pro-inflammatory macrophages and promoting M2 anti-inflammatory polarization [20,21].

Given the pivotal role of AKG in energy metabolism, nitrogen balance, intestinal health, and immune regulation, dietary AKG supplementation may represent an effective strategy to support the growth and health of weaned piglets. However, comprehensive evaluations of its effects on growth performance, diarrhea incidence, plasma amino acid profiles, and nutrient digestibility remain limited. Therefore, the objective of this study was to investigate the effects of dietary AKG supplementation on growth performance, diarrhea incidence, plasma amino acid concentrations, and fecal apparent nutrient digestibility in weaned piglets.

## 2. Materials and Methods

### 2.1. Animal Ethics Approval

The trial was conducted at the Langfang Experimental Farm of Hebei Province, China. The animal procedures in this study were approved by the Animal Care and Use Committee of the Institute of Feed Research of the Chinese Academy of Agricultural Sciences (IFR-CAAS20240302).

### 2.2. Experimental Design

A total of 72 weaned barrows (Duroc × Landrace × Yorkshire) with similar initial body weight (BW, 7.33 ± 0.50 kg) and age (28 ± 2 days) were randomly assigned to 3 treatments with 6 replicates per treatment and 4 pigs per pen, and balanced for BW and litter of origin. All the piglets were purchased from a Langfang commercial farm and housed in a nursery room. The experiment lasted for 42 days. The control group was fed a corn–soybean meal basal diet, while the AKG1 and AKG2 groups received the same basal diet with the addition of 500 g/t and 1000 g/t of alpha-ketoglutaric acid, respectively (supplied by Sinovac Biotech Ltd., Shenzhen, China). The amino acid content in the diet was adjusted by supplementing crystalline amino acids (lysine, methionine, threonine, tryptophan, valine, and isoleucine) to meet the nutritional requirements of piglets. The initial temperature of the facility was set at 28 °C and decreased by 1 °C per week until reaching 26 °C. Relative humidity was maintained between 55% and 65%. Throughout the experiment, piglets had *ad libitum* access to feed and water and were vaccinated following routine protocols, and the pig house was cleaned regularly. The experimental feed was prepared according to the nutritional requirements of NRC (2012) [22], and the feed formula and nutritional contents are shown in Table 1.

### 2.3. Growth Performance and Diarrhea Incidence

The body weights of piglets were recorded on days 0, 14, 28, and 42 of the experiment. Daily feed intake was recorded by weighing the residual feed in the troughs. Average daily gain (ADG), average daily feed intake (ADFI), and feed conversion ratio (FCR) were calculated accordingly. When pigs died, the weight of the pigs and the residual feed weight in the trough were recorded for the correction of growth performance data.

Diarrhea incidence was monitored daily from days 1 to 14. Each piglet was individually assessed at 09:00 a.m. by observing perianal swelling and the presence of loose feces in the trough. A 5-point fecal scoring system was used for visual assessment and monitoring of each piglet every day: 1 point = hard, granular feces; 2 points = hard, formed feces; 3 points = soft, formed feces; 4 points = soft, unformed feces; 5 points = watery feces. Feces in liquid form (4–5 points) were considered diarrhea.

### 2.4. Apparent Digestibility of Nutrients

On days 40, 41, and 42, feces were collected for three consecutive days. Diets and feces samples were analyzed for moisture (method 930.15) (AOAC, 2005) [23], ether extract (method 920.39A) (AOAC, 2005), and crude protein (N × 6.25; method 990.03) (AOAC, 2005). Gross energy (GE) was determined using a Parr 6400 calorimeter (Parr Instrument Company, Moline, IL, USA). The apparent digestibility of nutrients was calculated using acid-insoluble ash (AIA) as an internal marker. The AIA content in the experimental diets and feces was determined according to the method described by (Newkirk et al., 2003) [24]. The apparent digestibility of crude protein, ether extract, and gross energy in feces was determined by the endogenous indicator method.

### 2.5. Plasma Biochemical Indices

On days 14 and 42, one piglet with a body weight close to the average was selected from each pen for anterior vena cava blood collection. The disposable blood collection needle and anticoagulant blood collection vessel containing heparin sodium were used. After blood collection, it was reversed and mixed evenly. It was left at room temperature for 30 min, then centrifuged for 15 min at 3000 rpm, and plasma biochemical indicators (ALB, ALP, ALT, AST, GLU, HDL, LDL, TC, TG, TP) were measured using an HTSH-8000 biochemical analyzer (Hitachi Ltd., Tokyo, Japan).

### 2.6. Plasma Amino Acid Level

Plasma was pretreated with 1.5 M perchloric acid and 2 M potassium carbonate, centrifuged at 10,000 rpm for 10 min to obtain supernatant and then filtered by a 0.22 μm filter membrane. The contents of 16 amino acids (Asp, Glu, Ser, His, Gly, Thr, Arg, Ala, Tyr, Val, Met, Trp, Ile, Phe, Lys, and Leu) were analyzed by Shimadzu SIL-20A high-performance liquid chromatography (Shimadzu Corporation, Kyoto, Japan). A C18 reversed-phase HPLC column (5 μm, 100 A, 4.6 × 150 mm) was used for the separation. We determined the amino acid analysis following the recommended methods [25].

### 2.7. Statistical Analysis

The data related to growth performance, digestibility, plasma biochemical indices, and amino acid levels were analyzed by one-way ANOVA using the SAS 9.4 (SAS Institute Inc., Cary, NC, USA). Data are presented as means ± SEM. The dose-related effect of AKG was computed by GLM, using contrast command for the linear and quadratic effects. The chi-square test was utilized to analyze the incidence of diarrhea. Statistical significance was considered at *p* < 0.05, and a tendency was noted when 0.05 ≤ *p* < 0.10.

## 3. Results

### 3.1. Growth Performance and Diarrhea Incidence of Weaned Piglets

**Figure 1:**
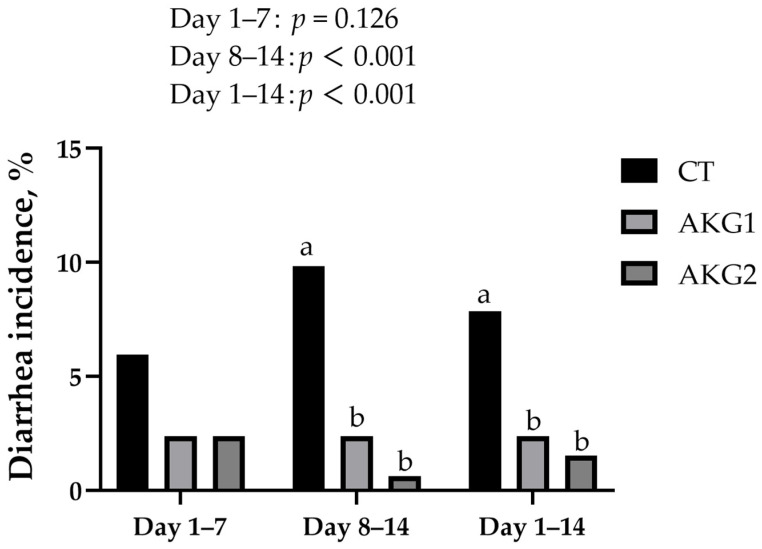
Effects of AKG on the diarrhea incidence of weaned piglets. ^a,b^ Values with different superscripts are significantly different (*p* ≤ 0.05).

Table 2 illustrates the influence of varying doses of AKG on growth performance in weaned piglets. Diets containing AKG had no significant effects on BW, ADG, ADFI, and FCR of weaned piglets. Figure 1 demonstrates the influence of varying doses of AKG on the diarrhea rate in weaned piglets. Compared with the control, feeding piglets with both 500 g/t and 1000 g/t AKG significantly reduced the diarrhea incidence of piglets (*p* < 0.05).

### 3.2. Nutrient Apparent Digestibility of Weaned Piglets

Table 3 shows the influence of alpha-ketoglutarate on fecal nutrient apparent digestibility of weaned piglets. The dry matter digestibility of weaned piglets consuming 1000 g/t AKG was significantly higher than that of 500 g/t (*p* < 0.05) group, and there was no significant change compared with the control group. As the AKG dose increases, the dry matter digestibility showed a significantly quadratic effect (*p* < 0.05), and the digestibility of crude protein and gross energy tended to increase (*p* = 0.056 and 0.054, respectively). In addition, the crude protein digestibility of weaned piglets in the AKG2 group tended to be higher than that in the CT group and AKG1 group (*p* = 0.092), while the energy utilization rate of piglets in the AKG2 group tended to be higher than that in the AKG1 group (*p* = 0.092).

### 3.3. Plasma Biochemistry Parameters of Weaned Piglets

Table 4 shows the influence of alpha-ketoglutarate on the fecal nutrient apparent digestibility of weaned piglets. The dry matter digestibility of weaned piglets consuming 1000 g/t AKG was significantly higher than that of the 500 g/t (*p* < 0.05) group, and there was no significant change compared with the control group. As the AKG dose increases, the dry matter digestibility showed a significantly quadratic effect (*p* < 0.05), and the digestibility of crude protein and gross energy tended to increase (*p* = 0.056 and 0.054, respectively). In addition, the crude protein digestibility of weaned piglets in the AKG2 group tended to be higher than that in the CT group and AKG1 group (*p* = 0.092), while the energy utilization rate of piglets in the AKG2 group tendend to be higher than that in the AKG1 group (*p* = 0.092).

### 3.4. Plasma Amino Acid Level of Weaned Piglets

Table 5 demonstrates the influence of AKG on the plasma amino acid level of weaned piglets. Compared with the control group, AKG had no significant effect on the plasma amino acid content of weaned piglets. The Phe content in the plasma of the AKG1 group tended to be lower than that of the CT group on day 14 (*p* = 0.089). On day 42, the Tyr content in the plasma of the AKG1 group tended to be lower than that of the CT group (*p* = 0.091). 

## 4. Discussion

Weaning-induced gut dysfunction in piglets may result from alterations in intestinal structure and the specific loss of digestive enzymes [26]. At weaning, the gastrointestinal system is still immature, with insufficient secretion of gastric acid and digestive enzymes, limiting the digestion of solid feed. Consequently, undigested proteins can enter the large intestine, deteriorate, and promote the proliferation of pathogenic bacteria, leading to diarrhea [27]. Consistent with previous findings, dietary supplementation with 1% AKG alleviated LPS-induced diarrhea in weaned piglets [28]. The present study demonstrated that supplementation with 500 g/t and 1000 g/t AKG significantly reduced diarrhea incidence in weanling piglets.

In this study, AKG supplementation improved nutrient digestibility. Previous research reported that 10 g/kg AKG supplementation increased the apparent N-digestibility and net protein utilization of growing pigs [29]. Similarly, higher trypsin activity was observed in juvenile mirror carp fed a diet containing 0.6% AKG [30]. As a precursor of glutamine, AKG provides both energy and nitrogen sources for intestinal epithelial cells, reduces intestinal glutamine catabolism, supports gastrointestinal cellular metabolism, and ensures normal nutrient absorption [31,32]. These findings suggest that AKG may reduce diarrhea incidence by enhancing nutrient digestibility in weanling piglets.

Most plasma proteins are synthesized by the liver, with ALB being a major component responsible for maintaining plasma osmolality and reflecting both liver function and immune status. It has been reported that supplementing a low-protein diet with 1% and 1.5% AKG significantly increased serum ALB concentrations in piglets [33]. In this study, plasma ALB content was significantly higher in the AKG2 group compared to the AKG1 group on day 42, displaying a quadratic dose–response pattern. This suggests that AKG supplementation may promote protein synthesis and metabolism in piglets. Serum ALP, primarily derived from the liver, serves as an important indicator of hepatobiliary function or bone metabolism. Previous studies reported that supplementation with 0.2–1.0% AKG increased serum ALP activity in juvenile mirror carp [34]. In our study, although the difference was not statistically significant, plasma ALP levels in the AKG2 group were numerically higher than those in the AKG1 group on day 14, showing a similar direction of change. However, He et al. (2007) observed that 1% AKG supplementation significantly reduced serum ALP content in piglets, suggesting that the specific effects of AKG on liver function require further investigation [35]. An inverse relationship between serum HDL-C concentrations and coronary heart disease risk is well established [36]. Radzki et al. (2009) reported that AKG supplementation (0.01 M and 0.1 M) increased HDL concentrations in rats with experimentally induced hypercholesterolemia. In our study, plasma HDL concentrations showed a linear increase with AKG dosage on day 42, indicating that AKG supplementation may have the potential to reduce cardiovascular disease risk and promote overall health in piglets [37]. AKG has also been shown to stimulate amino acid absorption while decreasing plasma glucose absorption, thereby alleviating hyperglycemia by inhibiting hepatic gluconeogenesis [38]. GLU serves as a major direct energy source in animals, generating ATP through glycolysis and the TCA cycle. In the present study, supplementation with 1000 g/t AKG significantly increased plasma GLU concentrations on day 42, exhibiting a quadratic dose–response effect, suggesting that this dose may be optimal for promoting energy metabolism in weanling piglets. 

In healthy animals, plasma amino acid (AA) concentrations are maintained within a relatively constant range. AA regulates key pathways essential for growth, development, immunity, and intestinal health, and serves as substrates for protein synthesis in intestinal mucosal cells [39]. He et al. (2016) observed that piglets fed diets supplemented with 1% AKG exhibited lower serum concentrations of Asp, Glu, Ala, Ile, Tyr, Phe, Lys, and Arg compared to controls, suggesting that AKG supplementation improves AA utilization [16]. Similarly, in our study, plasma concentrations of Phe on day 14 and Tyr on day 42 in the AKG1 group tendee to decrease and show a quadratic response, respectively, compared to the control group, although these differences were not statistically significant. Additionally, Phe activates the synthesis of BH4 and relates to neurological development and function, while Tyr participates in protein phosphorylation, nitrosation, and sulfation, and the degradation of both Phe and Tyr occurs primarily in the liver [40]. The observed decrease in Phe and Tyr values may reflect the effects of AKG supplementation on amino acid metabolism in piglets. Thus, these findings suggest that 500 g/t AKG may enhance the utilization of Phe and Tyr for tissue protein synthesis or promote their hepatic oxidation. However, most of these results showed positive trends, indicating certain limitations in our study, likely due to the small sample size. Therefore, future research will involve larger sample sizes and longer experimental durations to further validate these hypotheses.

## 5. Conclusions

In conclusion, dietary AKG showed no significant effect on the growth performance of piglets. However, both 500 g/t and 1000 g/t AKG significantly reduced the incidence of diarrhea in weaned piglets. Moreover, supplementation with 1000 g/t AKG improved fecal dry matter digestibility as well as plasma ALB and GLU concentrations on day 42. These findings suggest that AKG helps reduce diarrhea in piglets, potentially by promoting protein synthesis, enhancing energy metabolism, and improving overall health.

## Figures and Tables

**Table 1:** Ingredient composition of the diets (%, as-fed basis).

	Pre-Conservation Period			Late Conservation Period		
	CT	AKG1	AKG2	CT	AKG1	AKG2
Corn	46.46	46.46	46.46	60.3	60.3	60.3
Soybean meal, 46%	13.2	13.2	13.2	17.3	17.3	17.3
Expanded soybean	14.5	14.5	14.5	6.5	6.5	6.5
Fish meal	6	6	6	4	4	4
Whey powder	15	15	15	5	5	5
Soybean oil	1.1	1.1	1.1	1.5	1.5	1.5
Bran	0.112	0.112	0.112	0.02	0.02	0.02
Calcium dihydrogen phosphate	0.35	0.35	0.35	0	0	0
Limestone	0.77	0.77	0.77	0.6	0.6	0.6
Salt	0.4	0.4	0.4	1	1	1
Choline chloride, 60%	0.05	0.05	0.05	0.4	0.4	0.4
L-Lysine HCL, 65%	0.9	0.9	0.9	0.05	0.05	0.05
DL-Met	0.06	0.06	0.06	0.65	0.65	0.65
Threonine	0.24	0.24	0.24	0.05	0.05	0.05
Tryptophan	0.02	0.02	0.02	0.14	0.14	0.14
Valine	0	0	0	0.01	0.01	0.01
Isoleucine	0	0	0	0.6	0.6	0.6
Phytase (10,000)	0.02	0.02	0.02	0.02	0.02	0.02
Acidifier	0.2	0.2	0.2	0.2	0.2	0.2
Sodium butyrate	0.15	0.15	0.15	0.15	0.15	0.15
Alpha-ketoglutarate	0	0.05	0.1	0	0.1	0.2
Premix ^1^	0.448	0.448	0.448	0.268	0.268	0.268
	100.00	100.05	100.10	100	100.1	100.2
Nutrition composition						
Analyzed value						
CP, %	19.64	19.55	19.68	17.97	18.04	18.10
Ca, %	0.82	0.83	0.83	0.73	0.73	0.72
*p*, %	0.53	0.53	0.57	0.47	0.48	0.47
EE, %	4.34	4.65	4.72	5.92	6.35	6.45
Ash, %	5.82	5.84	5.84	11.20	11.02	11.18
Calculated value						
ME, kcal	3350	3350	3350	3300	3300	3300
Lys, %	1.25	1.25	1.25	1.22	1.22	1.22
Met, %	0.36	0.36	0.36	0.35	0.35	0.35
Thr, %	0.74	0.74	0.74	0.72	0.72	0.72
Trp, %	0.21	0.21	0.21	0.21	0.21	0.21

^1^ Premix supplied per kg of diet: vitamin A, 35.2 mg; vitamin D_3_, 7.68 mg; vitamin E, 128 mg; vitamin K_3_, 8.16 mg; vitamin B_1_, 4 mg; vitamin B_2_, 12 mg; vitamin B_6_, 8.32 mg; vitamin B_12_, 4.8 mg; Niacin, 38.4 mg; Calcium pantothenate, 25 mg; Folic acid, 1.68 mg; Biotin, 0.16 mg; Zn (ZnSO_4_ · H_2_O), 110 mg; Copper (CuSO_4_ · 5H_2_O), 125 mg; Iron (FeSO_4_ · H_2_O), 171 mg; Cobalt (CoCl_2_), 0.19 mg; Manganese (MnSO_4_·H_2_O), 42.31 mg; Iodine (Ca(IO_3_)_2_), 0.54 mg; Selenium (Na_2_SeO_3_), 0.19 mg.

**Table 2:** Effects of AKG on growth performance of weaned piglets.

	AKG Level, mg/kg				*p*-Value		
	0	500	1000	SEM	ANOVA	Linear	Quadratic
BW, kg							
Day 0	7.33	7.33	7.33	0.51	1.000	0.998	0.999
Day 14	11.75	11.45	12.03	0.77	0.862	0.796	0.635
Day 28	19.05	19.13	19.55	0.87	0.922	0.703	0.903
Day 42	27.81	27.97	28.48	1.12	0.835	0.552	0.991
ADG, g							
Day 0–14	315	294	336	24	0.469	0.555	0.282
Day 14–28	522	548	537	26	0.780	0.684	0.568
Day 28–42	626	632	638	30	0.675	0.402	0.783
Day 0–42	487	491	503	18	0.710	0.411	0.989
ADFI, g							
Day 0–14	432	398	443	29	0.633	0.754	0.369
Day 14–28	793	852	813	30	0.970	0.811	0.960
Day 28–42	1104	1115	1122	53	0.776	0.664	0.575
Day 0–42	777	788	793	30	0.913	0.673	0.963
FCR							
Day 0–14	1.394	1.372	1.319	0.052	0.656	0.398	0.730
Day 14–28	1.526	1.569	1.513	0.039	0.762	0.664	0.555
Day 28–42	1.763	1.770	1.759	0.030	0.440	0.291	0.471
Day 0–42	1.593	1.607	1.572	0.024	0.587	0.307	0.928

**Table 3:** Effect of AKG on the nutrient apparent digestibility (%) of weaned piglets.

	AKG Level, mg/kg				*p*-Value		
	0	500	1000	SEM	ANOVA	Linear	Quadratic
Dry matter	78.35 ^ab^	77.45 ^b^	79.70 ^a^	0.56	0.045	0.118	0.042
Crude protein	74.73	74.79	77.47	0.90	0.092	0.056	0.269
Ether extract	69.73	70.48	74.45	1.55	0.113	0.054	0.423
Gross energy	80.30	79.76	81.62	0.56	0.092	0.123	0.107

^a,b^ Means listed in the same row. Those with different superscripts are significantly different (*p* ≤ 0.05).

**Table 4:** Effects of AKG on the plasma biochemistry parameters of weaned piglets.

	AKG Level, mg/kg				*p*-Value		
	0	500	1000	SEM	ANOVA	Linear	Quadratic
Day 14							
ALB, g/L	22.13	21.52	23.24	1.07	0.537	0.481	0.393
ALP, IU/L	297	264	407	41	0.062	0.076	0.099
ALT, IU/L	49.50	55.26	51.12	4.27	0.640	0.797	0.370
AST, IU/L	87.96	91.15	82.38	10.45	0.852	0.726	0.665
GLU, mmol/L	3.42	3.74	3.53	0.41	0.862	0.852	0.614
HDL, mmol/L	0.40	0.51	0.51	0.05	0.208	0.116	0.412
LDL, mmol/L	0.90	1.05	1.00	0.08	0.494	0.451	0.363
TC, mmol/L	1.59	1.83	1.81	0.13	0.396	0.262	0.446
TG, mmol/L	0.74	0.68	0.68	0.09	0.899	0.702	0.808
TP, g/L	48.49	49.88	49.96	2.31	0.884	0.663	0.822
Day 42							
ALB, g/L	26.62 ^ab^	24.01 ^b^	30.79 ^a^	1.66	0.046	0.114	0.045
ALP, IU/L	200	199	249	21	0.199	0.123	0.352
ALT, IU/L	55.89	51.66	49.77	5.26	0.736	0.452	0.867
AST, IU/L	66.97	83.80	55.72	19.15	0.674	0.725	0.422
GLU, mmol/L	6.08 ^ab^	5.30 ^b^	6.39 ^a^	0.23	0.015	0.368	0.006
HDL, mmol/L	0.60	0.62	0.75	0.05	0.125	0.062	0.413
LDL, mmol/L	1.15	1.26	1.27	0.10	0.626	0.394	0.665
TC, mmol/L	2.08	2.24	2.36	0.12	0.341	0.151	0.886
TG, mmol/L	1.05	0.70	0.62	0.17	0.423	0.222	0.660
TP, g/L	62.76	60.04	63.12	1.76	0.454	0.892	0.219

^a,b^ Means listed in the same row. Those with different superscripts are significantly different (*p* ≤ 0.05).

**Table 5:** Effects of AKG on the plasma amino acid levels (mg/L) of weaned piglets.

	AKG Level, mg/kg				*p*-Value		
	0	500	1000	SEM	ANOVA	Linear	Quadratic
Day 14							
Asp	1.12	1.13	1.09	0.11	0.959	0.840	0.843
Glu	12.83	12.07	12.61	1.31	0.927	0.913	0.714
Ser	8.31	6.87	7.26	0.67	0.327	0.291	0.288
His	3.01	2.42	2.81	0.27	0.350	0.627	0.177
Gly	38.16	35.76	36.70	3.48	0.890	0.774	0.704
Thr	19.97	18.24	16.55	2.61	0.666	0.375	0.995
Arg	16.16	14.98	16.66	1.22	0.623	0.780	0.359
Ala	17.01	15.20	16.41	0.88	0.373	0.644	0.190
Tyr	6.36	5.99	6.64	1.25	0.935	0.876	0.745
Val	10.74	8.70	10.77	1.08	0.350	0.985	0.154
Met	5.16	5.17	4.97	0.37	0.917	0.726	0.830
Trp	3.15	2.31	2.98	0.43	0.393	0.785	0.187
Ile	8.57	7.28	7.80	0.95	0.663	0.594	0.470
Phe	5.66	3.95	4.87	0.49	0.089	0.289	0.051
Lys	18.94	16.74	15.82	1.40	0.423	0.209	0.761
Leu	9.30	7.17	8.75	1.25	0.505	0.774	0.265
Day 42							
Asp	1.16	1.06	1.18	0.11	0.752	0.911	0.462
Glu	14.04	13.03	13.94	1.52	0.878	0.963	0.617
Ser	6.05	5.69	5.97	0.53	0.886	0.922	0.636
His	6.29	7.09	6.44	0.73	0.722	0.892	0.434
Gly	36.27	33.03	36.45	3.11	0.693	0.968	0.400
Thr	13.49	10.78	10.40	1.30	0.284	0.150	0.519
Arg	10.72	10.19	10.25	0.94	0.920	0.744	0.815
Ala	22.68	20.41	20.30	1.68	0.611	0.385	0.646
Tyr	6.57	4.55	5.54	0.59	0.091	0.244	0.059
Val	9.42	9.15	9.57	0.86	0.949	0.909	0.768
Met	4.59	3.39	4.03	0.40	0.150	0.350	0.086
Trp	7.00	6.19	7.04	0.59	0.528	0.964	0.267
Ile	8.41	8.04	7.93	0.61	0.859	0.604	0.871
Phe	6.95	7.39	6.36	0.66	0.615	0.575	0.423
Lys	20.00	17.11	17.62	1.08	0.174	0.146	0.226
Leu	11.43	10.36	11.59	0.45	0.154	0.815	0.059

## Data Availability

Data are available on request from the authors.

## Abbreviations

The following abbreviations are used in this manuscript:

ADFI	Average Daily Feed Intake
ADG	Average Daily Gain
AKG	Alpha-ketoglutaric acid
Ala	Alanine acid
ALB	Albumin
ALP	Alkaline Phosphatase
Arg	Arginine acid
Asp	Aspartic acid
AST	Aspartate Aminotransferase
FCR	Feed Conversion Ratio
Glu	Glutamic acid
GLU	Glucose
Gly	Glycine acid
HDL	High-Density Lipoprotein
His	Histidine acid
Ile	Isoleucine acid
LDL	Low-Density Lipoprotein
Leu	Leucine acid
Lys	Lysine acid
Met	Methionine acid
Phe	Phenylalanine acid
Ser	Serine acid
TC	Total Cholesterol
TG	Triglycerides
Thr	Threonine acid
TP	Total Protein
Trp	Tryptophan acid
Tyr	Tyrosine acid
Val	Valine acid
